# A longitudinal evaluation of gastrointestinal symptoms in children with autism spectrum disorder

**DOI:** 10.1177/13623613251362349

**Published:** 2025-08-28

**Authors:** Bibiana Restrepo, Sandra L Taylor, Matthew Dominic Ponzini, Kathleen Angkustsiri, Marjorie Solomon, Sally J Rogers, Paul Ashwood, Daphne S Say, Sonny Caceres, Shayan Alavynejad, Brianna Heath, David G Amaral, Christine Wu Nordahl

**Affiliations:** 1 MIND (Medical Investigations of Neurodevelopmental Disorders) Institute, University of California, Davis, CA; 2 Division of Developmental and Behavioral Pediatrics, Department of Pediatrics, School of Medicine, University of California, Davis, CA; 3 Department of Public Health Sciences, School of Medicine, University of California, Davis, CA; 4 Department of Psychiatry and Behavioral Sciences, University of California Davis, Davis, CA; 5 Department of Medical Microbiology and Immunology, University of California, Davis, CA; 6 Division of Pediatric Gastroenterology, Department of Pediatrics, School of Medicine, University of California, Davis, CA; 7 Division of Internal Medicine, SUNY Downstate Health Sciences University, USA

**Keywords:** chronic GI, GI dysfunction, GI issues, GI symptoms, impaired behavior, longitudinal, medical problems

## Abstract

Gastrointestinal symptoms are frequently reported in children diagnosed with autism spectrum disorder. This study sought to determine the longitudinal trajectory of gastrointestinal symptoms without a medical etiology in children with autism compared to similar aged participants with typical development. A total of 475 children enrolled in this longitudinal study (322 autism spectrum disorder and 153 typical development groups) were evaluated at up to three time points between 2 and 12 years of age. Nine common gastrointestinal symptoms and formal medical gastrointestinal diagnosis were assessed using a physician-administered parent interview. A rigorous symptom classification was performed by physicians via clinical consensus. The frequency and persistence of gastrointestinal symptoms across childhood were compared between groups. Associations between gastrointestinal symptoms and measures of internalizing and externalizing behaviors, sleep problems, sensory problems, restricted and repetitive behaviors, and social communication were also evaluated. Children with autism presented with more gastrointestinal symptoms at each time point, and they were also more likely to experience multiple and persistent gastrointestinal symptoms. The presence and number of gastrointestinal symptoms were associated with greater impairment in internalizing behaviors, sleep, communication, sensory processing, and repetitive behaviors. Participants in the autism spectrum disorder group reported more gastrointestinal symptoms without known etiology throughout childhood in this longitudinal well-characterized sample.

**Lay Abstract:**

Children with autism have been found to experience more medical issues including gastrointestinal symptoms. In this study, participants in the autism group were more likely to experience gastrointestinal symptoms than their typically developing peers. They were also more likely to experience multiple gastrointestinal symptoms at the same time and more likely to have persistent gastrointestinal symptoms throughout their childhood. Increased gastrointestinal symptoms were associated with more challenges with sleep, communication, sensory processing, and repetitive behaviors. Clinicians and parents should become more aware of the high occurrence of gastrointestinal problems in children with autism. If identified, these symptoms are often treatable which may improve their well-being.

## Introduction

Autism spectrum disorder (ASD or autism) is a heterogeneous neurodevelopmental condition characterized by the presence of functionally impairing social communication challenges and restrictive, repetitive patterns of behavior (American Psychiatric Association, 2013). Autism has been identified in roughly 1 in every 36 children in the United States (Maenner et al., 2020). Children with autism often experience mental health and medical problems including sleep problems, seizure disorders, and gastrointestinal (GI) issues (Al-Beltagi, 2021; Neumeyer et al., 2019; Tye et al., 2019).

Although a consensus on the prevalence of gastrointestinal symptoms (GIS) in children with autism has not been established (Buie et al., 2010; Holingue et al., 2018; Leader et al., 2022), it is known that GI issues are one of the most common medical complaints (Aldinger et al., 2015; Doshi-Velez et al., 2014; Holingue et al., 2018; McElhanon et al., 2014; Valicenti-McDermott et al., 2006). GIS are more commonly reported in children with autism than peers with typical development (Chaidez et al., 2014; Mazurek et al., 2013; Restrepo et al., 2020; Valicenti-McDermott et al., 2006; Yang et al., 2018), with prevalence rates ranging from 4.2% to 96.8% with a median of 46.8% (Holingue et al., 2018). Frequently, these symptoms are not associated with a known underlying condition or formal GI diagnosis. Children with autism and GI disorders, including chronic constipation, have higher rates of hospitalizations (Aldinger et al., 2015; McMaughan et al., 2022) than children with typical development (TD) and their untreated symptoms have been linked to other medical, developmental, and behavioral problems (Aldinger et al., 2015; Ferguson et al., 2019). Previous studies have identified a relationship between GIS and the worsening and/or onset of problem behaviors in the population with autism (Ferguson et al., 2017, 2019; Maenner et al., 2012; Marler et al., 2017; Mazefsky et al., 2014). In our study cohort, we previously found that almost half of 2- to 3-year-old participants with autism experienced GIS, and that the presence of GIS was associated with increased self-injurious and aggressive behaviors as well as sleep and attention problems (Restrepo et al., 2020).

The relation between GI problems and autism is not completely understood. Some researchers have proposed that GIS are associated with food selectivity (Valenzuela-Zamora et al., 2022) and other behaviors frequently observed in children with autism (Hung & Margolis, 2024), including sensory selectivity (Cermak et al., 2010) and adherence to routines linked to selective eating and unbalanced diets (Valenzuela-Zamora et al., 2022). Other frequent findings such as social communication challenges and social compliance (Cumine et al., 2009) may also influence their feeding patterns (Byrska et al., 2023). There is a body of literature associating psychological factors such as internalizing, externalizing, and somatic complaints to GIS (Ferguson et al., 2019). Similarly, factors affecting the gut–microbiota–brain axis through the *enteric* nervous system can have a role (Hung & Margolis, 2024). Although these are plausible mechanistic pathways, this remains an area for further research.

To date, very few studies have examined the longitudinal course of GIS in children with autism (Black et al., 2002; Ibrahim et al., 2009; Mannion et al., 2013; Mazurek et al., 2014; Mouridsen et al., 2010; Sandhu et al., 2009). Two published studies (Mannion et al., 2013; Mazurek et al., 2014) tracked the persistence of GIS in this population at 1- and 2-year follow-up; they reported a significant persistence of symptomatology from baseline, while new symptoms developed in some individuals ages 2 to 17 years. Other studies (Black et al., 2002; Ibrahim et al., 2009; Mouridsen et al., 2010; Sandhu et al., 2009) have investigated the incidence of GI concerns between individuals with and without autism by analyzing retrospective medical chart data across the lifespan. These studies found no significant difference in incidence of GIS between these two groups during early life (Black et al., 2002; Sandhu et al., 2009), childhood and adolescence (Ibrahim et al., 2009), and adulthood (Mouridsen et al., 2010). However, significant limitations of the studies, including lack of a consistent GIS data collection, likely impacted the generalizability of findings.

To our knowledge, there are no prospective longitudinal studies that assess the frequency and persistence of GIS in children with autism throughout childhood using a well-characterized sample assessed using a comprehensive battery of standardized developmental and behavioral measures. The objective of the current study is to perform a longitudinal evaluation of the frequency and persistence of GIS with unknown etiology in participants in the ASD group compared to TD group. Building from our prior, cross-sectional study in preschoolers, we assessed GIS and behavioral measures at two additional time points later in childhood. We hypothesized that (1) compared to TD children, GIS would be more frequently reported and persistent in the ASD group across childhood and (2) the presence of GIS would be associated with more severe impairments across several behavioral domains throughout childhood.

## Methods

This study was approved by the University of California Davis Institutional Research Board (IRB) and informed consent was obtained from the parent/guardian of each participant. Participants were recruited from the community via social media posts, fliers placed in public gathering places, pediatrician offices, and community health fairs. Participants were also recruited from our institution Participant Research Registry. Participants are enrolled in the UC Davis MIND Institute Autism Phenome Project (APP), an ongoing, multidisciplinary longitudinal study. Enrolled participants were assessed at up to four time points: baseline (2–4 years; Visit 1), 2 years later (4–6 years; Visit 3), and again during middle childhood (9–12 years; Visit 4). The APP includes a Visit 2 which consists of only neuroimaging data collection and is thus omitted from the present study. A subset of this dataset was utilized in our previous cross-sectional study of GIS at T1 (Restrepo et al., 2020). All participants were English speaking, ambulatory, and had no severe motor, vision, hearing, or diagnosed chronic health problems that would preclude their ability to complete the assessment protocol required for this study. TD participants were screened for autism using the Social Communication Questionnaire (SCQ; Rutter, Bailey, & Lord, 2003, and excluded if scores were ⩾ 11) or if they had first-degree relatives with ASD. TD participants were also excluded for developmental delay if developmental quotient was below 70 on the Mullen Scales of Early Learning (MSEL) (Mullen, 1995). At T1, ASD was confirmed using the Autism Diagnostic Observation Schedule (ADOS-Generic or ADOS-2) (Lord, 1999; Luyster, 2012) and the Autism Diagnostic Interview–Revised (ADI-R; Lord et al., 1994; Rutter, Le Couteur, & Lord, 2003) conducted by research reliable licensed clinical psychologists. For children in the ASD group, ASD diagnoses were confirmed at the later time points by a licensed psychologist using the ADOS-2 and *Diagnostic and Statistical Manual of Mental Disorders* (DSM) checklist to evaluate the stability of the diagnosis. All participants in the ASD group met diagnostic criteria ASD at all time points.

Participants were evaluated with a battery of diagnostic, medical (GIS), developmental, and behavioral measures collected at the time of enrollment and subsequent time points as part of a comprehensive interdisciplinary assessment as follows.

### Assessment of gastrointestinal symptoms

At each time point, caregivers were interviewed by developmental and behavioral pediatricians specializing in autism using a gastrointestinal history questionnaire that has been utilized by the Childhood Autism Risks from Genetics and Environment (CHARGE) study to assess GIS in children with autism (CHARGE-GH) (Chaidez et al., 2014; Restrepo et al., 2020). Specifically, the CHARGE-GH assesses the presence and frequency of nine common GIS: abdominal pain, gaseousness/bloating, diarrhea, constipation, pain on stooling, vomiting, difficulty swallowing, blood in stool, and blood in vomit. Frequency of each symptom is rated on a 5-point Likert-type scale (0 = never, 1 = rarely, 2 = sometimes, 3 = frequently, 4 = always). “Current” symptoms were defined as those experienced during the last 3 months and “Previous” if the symptoms were endorsed prior to the last 3 months. Participants missing information on more than three symptoms at any time point were coded as missing. In addition, there were three open-ended questions exploring any underlying known GI diagnosis, diagnosed food allergies, and food intolerance or dietary restrictions due to worsening GIS.

Participants experiencing at least one current GI symptom in the “sometimes,” “frequently,” or “always” range were categorized as having GIS (ASD-GI or TD-GI), and those reporting symptoms in the “never” or “rarely” ranges were classified as ASD-noGI and TD-noGI groups.

Because the goal of this study is to evaluate the frequency, persistence, and effect of GIS without a known etiology, participants with an underlying medical GI diagnosis explaining symptomatology were not included in the GI group. Established GI diagnosis for those in this group included gastroesophageal reflux disease (ASD = 12, TD = 2), chronic gastritis (ASD = 1), malabsorption (ASD = 2), colitis (ASD = 2), celiac disease (ASD = 2), and possible irritable bowel syndrome (ASD = 1). Three of the participants in the ASD group had two diagnoses. Participants whose parents reported food allergies (ASD = 43, TD = 11) or dietary restrictions due to food intolerance, defined as GIS elicited upon specific food consumption (ASD = 67, TD = 14), were not considered to have unexplained GIS and thus were not classified into GI group. The characterization of presence or absence of GIS based on the CHARGE-GH was performed by two licensed physicians independently (B.R., S.C.). In addition, coding reliability was ensured via weekly meetings by the coding physicians reviewing and discussing coding based on criteria or symptom definition to clinical consensus to collectively determine GI classification. A pediatric GI specialist was available as needed for consensus (D.S.S.).

### Developmental and behavioral measures

We included longitudinal behavioral and developmental information previously analyzed in available literature and in our previous cross-sectional study. Parent questionnaires were completed by caregivers who reside with the participant and have knowledge of the child’s behaviors. Supplemental Table 1 provides a breakdown of specific caregivers (i.e. mother, father, grandparent) responding to study questionnaires.

The assessment of cognitive abilities was obtained by using either the MSEL (Mullen, 1995) or the Differential Abilities Scales-II (DAS-II) (Elliot, 2007). The MSEL measures cognitive and developmental functioning from infancy and up to 68 months of age in a standardized manner. Verbal, non-verbal, and combined IQ were estimated by calculating ratio DQ scores. The DAS-II assesses children’s cognitive abilities between 2.5 and 17 years of age in a standardized manner.

The Parent/Caregiver Rating Form for Vineland Adaptive Behavior Scales, Second Edition (VABS-2) was completed by caregivers to assess adaptive domains yielding age-referenced standard adaptive behavior composite score (Sparrow et al., 1985). Standard scores from the subscales of communication, daily living skills, motor, and social domains as well as the adaptive behavior composite score were utilized.

Parents/caregivers also completed the Repetitive Behavior Scale–Revised (RBS-R) questionnaire that evaluates the severity of restrictive and repetitive behaviors in autism (Bodfish, 2003; Bodfish et al., 2000). We utilized scores from a three-factor solution (Mirenda et al., 2010) that includes Compulsive/Ritualistic/Sameness Behaviors (CRSB), Self-Injurious Behaviors (SIB), and Restrictive Stereotyped Behaviors (RSB) as well as a total score.

The Social Responsiveness Scale-2 preschool version (SRS), a 65-item parent-rated instrument (Constantino et al., 2000, 2003), was also completed. The Social Communication and Interaction score and Restrictive and Repetitive Behavior subscale scores, as well as the total score, were used in the current analyses. The occurrence of sensory processing difficulties was assessed using the Short Sensory Profile, version 1 (SSP-1) to assess sensory processing in children (Dunn, 1999). We used a shortened form of the Dunn’s Sensory Profile caregiver questionnaire (McIntosh et al., 1999). The SSP-1 subscale scores (Auditory Filtering, Low Energy/Weak, Movement Sensitivity, Tactile Sensitivity, Taste/Smell Sensitivity, Under-responsive/Seeks Sensation, and Visual/Auditory Sensitivity) as well as the total score were utilized. SSP-1 data was only available at Visits 1 and 3. For Visit 4, the SSP-2 was collected instead. Because SSP-1 and SSP-2 data cannot be combined, we elected to utilize only SSP-1 data from Visits 1 and 3.

The Child Behavior Checklist (CBCL)-Preschool (Achenbach & Rescorla, 2000) and School (Achenbach, 1991) versions were used to assess current behavioral, social, and emotional problems. Standard scores for syndrome scales were used (Emotionally Reactive, Anxious/Depressed, Somatic Complaints, Withdrawn, Sleep Problems, Attention Problems, and Aggressive Behavior): Internalizing and externalizing scales were also included (Achenbach & Ruffle, 2000).

The Children’s Sleep Habits Questionnaire (CSHQ), a 45-item parent questionnaire, was also included to address sleep behaviors in children aged 2–10 during the last month (Goodlin-Jones et al., 2009; Owens et al., 2000). Subscale scores (Bedtime Resistance, Sleep Onset Delay, Sleep Duration, Sleep Anxiety, Night Waking, Parasomnias, Sleep Disordered Breathing, Daytime Sleepiness) as well as the total score were utilized in the analyses.

### Analytic plan

#### Subgrouping based on the presence or absence of GIS

First, we describe the proportion of children in each group reporting GIS at each time point and those with persistent GIS at two or more time points. A chi-square test was used to test differences in the occurrence of persistent GIS by group.

#### Relative risk across time points

Next, to evaluate whether children with autism had more GIS at each time point than TD participants, we fitted a mixed effect log binomial regression to model the relative risk (RR) of GIS over time. Predictors included group (ASD, TD), sex (F, M), and age (years). Age was the average of the ages reported when CHARGE-GH, CBCL, and ADOS assessments were administered. We first fit a full model that included all main effects, two-way and three-way interactions with a random subject effect to account for within-subject correlation. If interactions were not significant, a model with just the main effects of group, age, and sex was utilized. A robust covariance structure was used to ensure adequate confidence interval coverage.

#### GI symptom frequency, as indexed by the number of concurrent GIS, across time points

To assess whether the ASD group experienced a greater number of GIS at each time point and across time than the TD group, we fit a mixed effect Poisson regression. Main effects and all two- and three-way interactions between group, sex, and age were evaluated. A main effects only model was then evaluated if no interactions were statistically significant. A random intercept was included to account for within-subject correlation and a robust covariance structure was employed.

#### GI symptom subgroups and behavioral scores across time points

To assess the effect of the presence or absence of GIS on behavioral scores over time, we used linear mixed effects models. Each behavioral score was first modeled versus the presence of GIS at each time point (Y/N), sex, age at each visit, group, and all two-way interactions between these factors. IQ was included as a covariate and a random subject effect was included to account for within-subject correlation. To account for multiple testing across behavior models, we calculated false discovery rates (FDRs) for each predictor/interaction term and evaluated significance based on FDR < 0.05. If interaction terms with GIS were not significant for any behavioral score (FDR > 0.05), we reduced models to include only main effects.

#### GIS frequency, as indexed by number of concurrent GIS, and behavioral scores

We also evaluated the effect of having multiple concurrent GIS on behavioral scores. As few TD children had multiple GIS, we restricted these analyses to the ASD group. Linear mixed effect models were used to model each behavioral score versus the number of GIS, sex, age, and all two-way interactions among these plus IQ as a covariate. A random intercept was included to account for within-subject correlation. Significance was evaluated based on FDR values (<0.05) calculated across all behavior models for each factor and interaction term. If no interaction terms with GIS severity were significant (FDR > 0.05), we reduced models to include only main effects.

## Results

### Participant characteristics

A total of 475 children are included in this study (322 ASD and 153 TD). Participants enrolled in the APP were included if they had at least one time point with complete GI data. Table 1 reports participant characteristics at each time point. Groups did not differ by age at any time point. The TD group had higher DQ/IQ scores than the ASD group at each time point.

**Table 1.** Participant characteristics.

	ASD	TD
n (Female)	322 (96)	153 (67)
Number of participants at each visit: n		
Visit 1	316	151
Visit 3	91	53
Visit 4	77	51
Age (years): Mean (SD)		
Visit 1	3.1 (0.5)	3.0 (0.6)
Visit 3	5.7 (1.0)	5.8 (1.1)
Visit 4	11.5 (1.0)	11.6 (0.8)
ADOS CSS: Mean (SD)		
Visit 1	7.5 (1.7)	–
Visit 3	7.1 (2.1)	–
Visit 4	7.5 (2.0)	–
Full Scale IQ: Mean (SD)		
Visit 1	63.4 (20.8)	106 (11.9)
Visit 3	79.9 (32.3)	113.3 (10.8)
Visit 4	74.6 (32.8)	113.1 (13.4)
Total number of GI assessments: n (%)		
1	200 (62.1%)	80 (52.3%)
2	82 (25.5 %)	44 (28.8%)
3	40 (12.4%)	29 (19.0%)

At Visit 1, Development Quotient (DQ) is reported instead of IQ; ADOS-CSS: Autism Diagnostic Observation Scale Calibrated Severity Score. This diagnostic assessment was not administered to participants in the TD group.

**Figure 1.**
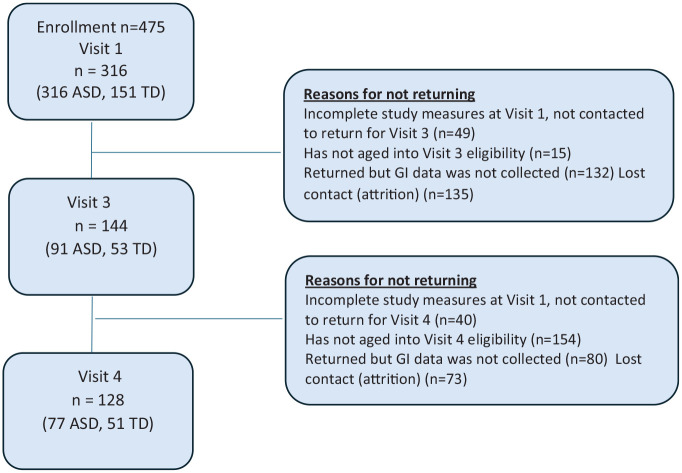
This consort diagram illustrates the study retention and attrition throughout the different time points. Measures required for inclusion in this analysis (GI questionnaire, developmental and behavioral measures). Visit 2 is not depicted because GI measures were not acquired at that time point and therefore not included in this study. Reasons for not returning at follow-up visits included (1) participant is too young to participate in visit, (2) did not complete study measures, and (3) study unable to get in contact with parents or declined participation.

Figure 1 depicts the return rates and reasons for missing data at later time points in this longitudinal study. The study is ongoing and includes a larger battery of assessments than are included in the current analyses. Participants were only asked to return at later time points if they had completed all of the initial study components. This accounts for some of the missing data at later time points (n = 49 at Visit 3 and n = 40 at Visit 4). In addition, given that data collection is ongoing, there is a substantial proportion of participants that have not yet aged into the eligible age range of the longitudinal visits, particularly for the later middle childhood time point (n = 15 at Visit 3 and n = 154 at Visit 4). A third reason for missing data is that some participants returned for other components of the study, but GIS data was not collected due to scheduling conflicts with either the family or the pediatrician (n = 132 at Visit 3 and n = 80 at Visit 4). We have separated out these three reasons from true attrition, which is defined as lost contact with the family or declined to participate (n = 134 at Visit 3 and n = 73 at Visit 4).

We have also conducted a supplemental analysis comparing participants in the study with those lost to attrition and those who returned but GI data was not collected at Visit 3 and Visit 4 for ASD and TD participants (see Supplemental Tables 2 and 3). For both ASD and TD groups, there were no significant differences between these three groups (Returned with GI; Returned, missing GI; attrition) for proportions endorsing GI symptoms or IQ scores. For participants in the ASD group, there was no significant differences in ADOS CSS scores across these three groups at Visit 3 (p = 0.206), but there was a trend toward an overall group differences in ADOS-CSS scores at Visit 4 (p = 0.055); the participants who were lost to attrition to have slightly higher ADOS-CSS scores compared to those who returned and had GI data (8.5 vs 7.0).

*Hypothesis 1.* Frequency of GIS across groups.

#### Subgrouping based on the presence or absence of GIS

**Figure 2.**
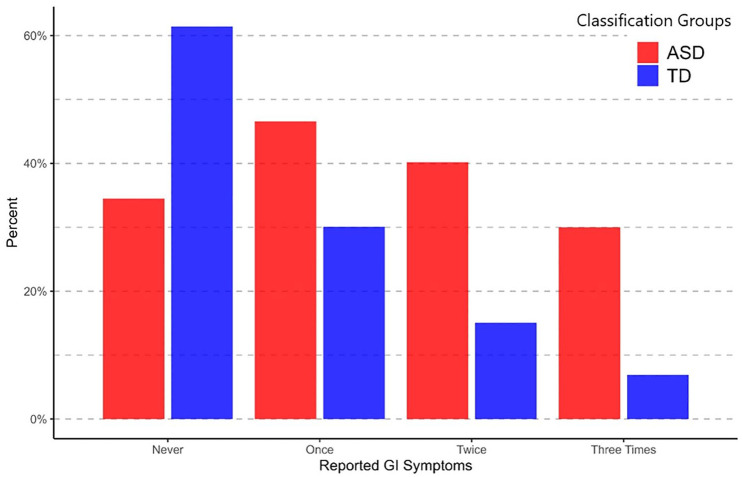
Percentage of ASD and TD participants who never reported GIS or reported GIS once, twice, or three times. While majority of TD participants never reported GIS, 30% of children with ASD reported GIS at all three visits.

Figure 2 depicts the percentage of participants in each group (ASD and TD) that endorsed GIS at 0, 1, 2, or 3 visits (i.e. never, once, twice, or three times). The percentage of children in the TD group never reporting GIS was nearly twice that of the ASD group (61.4% vs 34.5%). Moreover, a higher percentage of participants in the ASD group reported GIS across all time points as illustrated in Figure 2.

Table 2 provides additional detail about the percentage of participants who endorsed GIS at each specific visit or combination of visits. For example, for participants in the ASD group who reported GIS at only one visit, 37.3% reported GIS at Visit 1, 20.9% reported GIS at Visit 3, and 16.9% reported GIS at Visit 4. The numerator and denominator of each percentage is determined by the number of participants with GI data at each visit or combination of visits. As seen in Table 2, many participants only had GIS reported at one visit ASD = 46.6% (n = 150), TD = 27.7% (n = 46). Children with autism were much more likely than children in the TD group to report symptoms at two visits (40.2% vs 15.1%) or all three (30.0% vs 6.9%) visits. Similarly, the ASD group experienced more persistent GIS (defined as two or more visits). Among children with GI data for at least two visits, we found that 50% of the children with autism (*n* = 61) had persistent GIS. This percentage was statistically significantly greater than for children in the TD group with only 13 (18%) having persistent GIS (χ^2^ = 18.8, p < 0.001).

**Table 2.** Number and percentages of ASD and TD children reporting GIS across visits.

	ASD	TD
**Never reported GIS**	**111/322 (34.5%)**	**94/153 (61.4%)**
**GIS at exactly one visit**	**150/322 (46.6%)**	**46/153 (30.1%)**
Visit 1	118/316 (37.3%)	33/151 (21.9%)
Visit 3	19/91 (20.9%)	5/53 (9.4%)
Visit 4	13/77 (16.9%)	8/51 (15.7%)
**GIS at exactly two visits**	**49/122 (40.2%)**	**11/73 (15.1%)**
Visits 1, 3	22/87 (25.3%)	4/53 (7.6%)
Visits 1, 4	20/75 (26.7%)	5/49 (10.2%)
Visits 3, 4	7/40 (17.5%)	2/29 (6.9%)
**GIS at all three visits**	**12/40 (30%)**	**2/29 (6.9%)**

Denominators differ based on the number of participants with GI data at the indicated time points.

At the symptom level, constipation, diarrhea, gaseousness/bloating, and abdominal pain were the most common symptoms in the ASD group. For participants in the TD group, similar symptoms were reported but at lower rates. Table 3 depicts frequency of each GIS reported across all time points. Supplemental Figure 1 depicts the overlap between GIS at each time point.

**Table 3.** Frequency of individual GIS across all participants and time points.

	ASD	TD
Constipation	32.4%	11.3%
Diarrhea	26.7%	11.2%
Gaseousness/bloating	26.5%	11.3%
Abdominal pain	16.6%	11.7%
Pain on stooling	17.6%	5.9%
Vomiting	6.2%	4.3%
Difficulty swallowing	5.4%	0%
Blood in stool	1.9%	0.8%
Blood in vomit	0%	0%

#### Relative risk across time points

**Figure 3.**
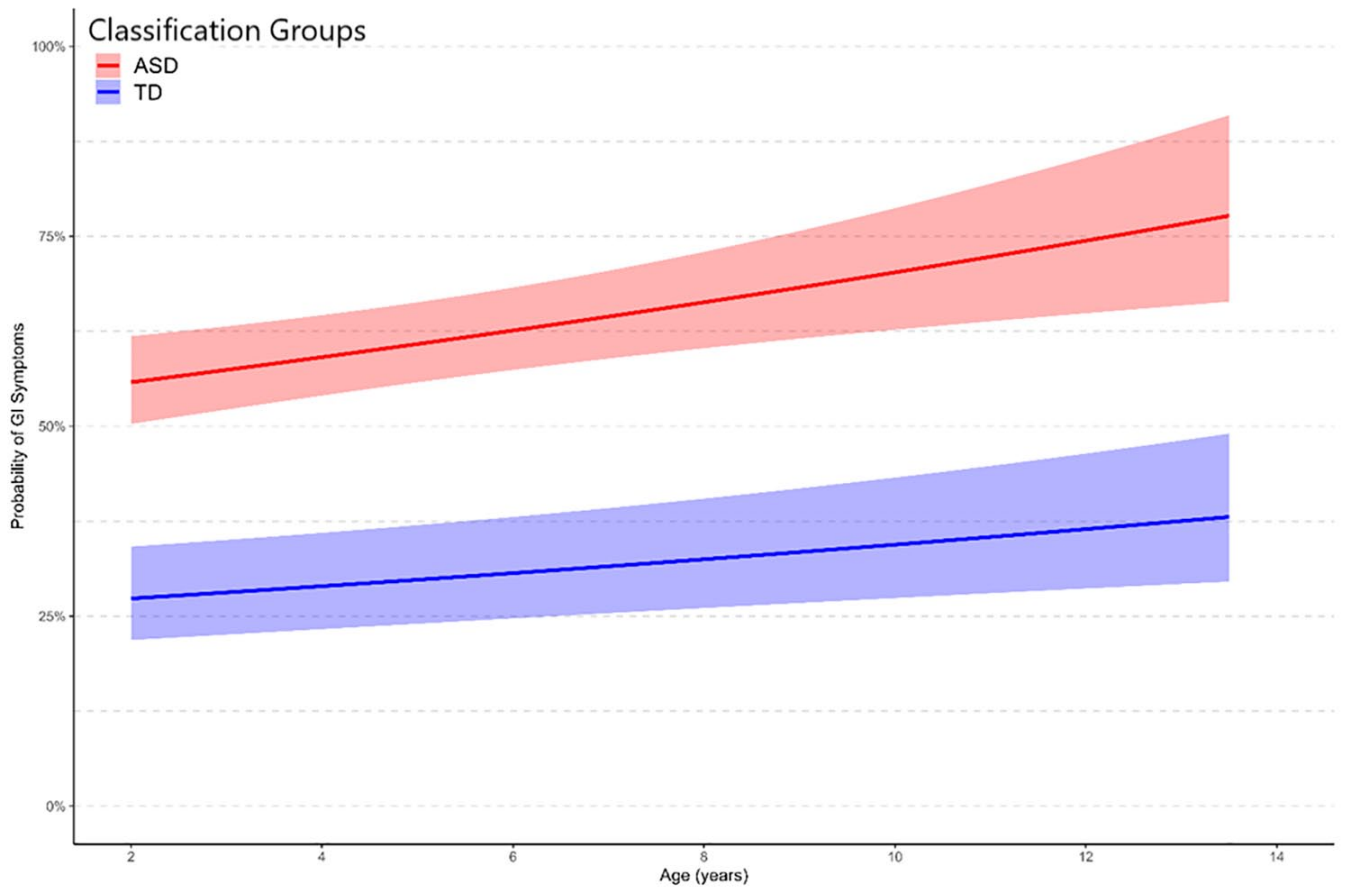
Predicted probability of GIS over time for participants in ASD and TD groups.

Overall, a higher proportion of children with autism experienced GIS at each time point and at multiple time points. The occurrence of GIS is significantly higher for children in the ASD group with a RR of GIS in ASD to TD groups of 2.04 [1.62, 2.57] (t_260_ = = 3.46, p < 0.0001) and increases for every 1-year increase in age (RR = 1.03 [1.01, 1.05]) (t_260_ = 3.46, p = 0.0006)) as illustrated in Figure 3. Although not achieving statistical significance at 0.05, the estimated risk of GIS was higher in females than males (RR = 1.15 [0.98, 1.36]) (t_260_ C = 1.73, p = 0.0854).

#### GI symptom frequency, as indexed by the number of concurrent GIS, across time points

On average, children with autism reported 2.4 [1.9, 3.1] more concurrent GIS than TD children (t_263_ = 7.16, p < 0.001). Across both groups, females averaged 1.4 [1.1, 1.7] more GIS than males (t_263_ = 2.74, p = 0.007). The number of symptoms reported increased slightly over time, with the number of symptoms reported increasing by 3.7% [1.4%, 6.1%] per year on average (t_263_ = 3.14, p = 0.002).

*Hypothesis 2*. Association between the presence of GIS and impaired behaviors.

#### GI symptom subgroups and behavioral scores across time points

**Figure 4.**
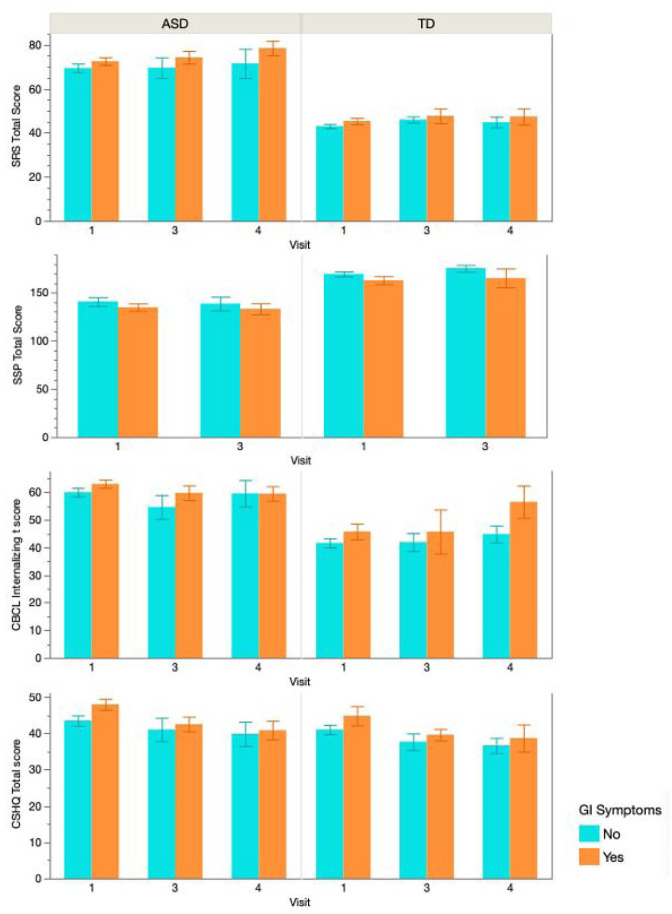
Observed mean scores (95% confidence limits) for behavioral measures that differed significantly in participants with and without GI symptoms (see Table 4 for FDR-corrected p-values). Across both ASD and TD groups, children with GIS had more severe scores on each of these measures. SSP-1 data was not available at Visit 4.

We found significant associations between the presence of GIS and scores that reflected greater impairment for social communication/interaction (p = 0.003); total score for SRS (p = 0.005); auditory filtering (p = 0.016) and visual/auditory sensitivity for the SSP-1 (p = 0.006); anxious-depressed symptoms (p = 0.008), internalizing behaviors (p = 0.0009), sleep problems (p = 0.02), and somatic complaints (p < 0.0001) scores in the CBCL; and daytime sleepiness (p = 0.0009), parasomnia (p = 0.003), and total score (p = 0.003) in the CSHQ (Table 4). There were no significant differences on any of the VABS scores. Figure 4 shows the difference in total scores for the various measures, between children with and without GIS for the ASD and TD groups. Full model results are provided in Supplementary Tables 4(a) and (b).

**Table 4.** Association between the presence of GI symptoms and behavioral scores.

	Estimate (SE)	p	FDR
**Repetitive Behavior Scale Total Score**	1.79 (1.13)	0.1155	0.1826
Compulsive/ritualistic/sameness behaviors	1.37 (0.76)	0.0727	0.1369
Self-injurious behaviors	0.26 (0.21)	0.2162	0.2863
Restrictive stereotyped behaviors	0.23 (0.35)	0.5034	0.5434
**Social Responsiveness Scale-2 Total Score**	2.47 (0.73)	0.0009	**0.0051**
Repetitive and restrictive behaviors	1.97 (0.87)	0.0245	0.0763
Social Communication and Interaction	2.55 (0.71)	0.0004	**0.0031**
**Short Sensory Profile-1 Total Score**	-5.69 (1.73)	0.0014	**0.0063**
Auditory filtering	-1.14 (0.39)	0.0045	**0.0167**
Low energy/weak	-0.84 (0.44)	0.0609	0.1237
Movement sensitivity	-0.05 (0.21)	0.8225	0.847
Tactile sensitivity	-0.67 (0.37)	0.0779	0.1395
Taste/smell sensitivity	-0.84 (0.44)	0.0593	0.1237
Under-responsive/seeks sensation	-1.09 (0.52)	0.039	0.1005
Visual/auditory sensitivity	-1.26 (0.38)	0.0013	**0.0063**
**Child Behavior Checklist**			
Externalizing problems t-score	1.35 (0.81)	0.0992	0.1679
Internalizing problems t-score	3.12 (0.77)	<0.0001	**0.0009**
Aggressive behavior	0.43 (0.63)	0.496	0.5434
Anxious/depressed	1.46 (0.47)	0.002	**0.0081**
Attention problems	0.43 (0.6)	0.4739	0.5378
Defiant behavior	1.13 (0.86)	0.2152	0.2863
Emotionally reactive	1.27 (0.66)	0.0569	0.1237
Sleep problems	2.01 (0.73)	0.0071	**0.0242**
Social problems	1.68 (1.19)	0.1858	0.2632
Somatic complaints	4.17 (0.53)	<0.0001	**<0.0001**
Thought problem	1.23 (1.29)	0.3657	0.4298
Withdrawn	0.38 (0.68)	0.5755	0.6056
**Children’s Sleep Habit Questionnaire Total Score**	2.34 (0.65)	0.0005	**0.0031**
Bedtime resistance	0.27 (0.27)	0.3286	0.4078
Disordered breathing	0.1 (0.05)	0.0418	0.1005
Daytime sleepiness	1.07 (0.26)	<0.0001	**0.0009**
Night waking	0.27 (0.13)	0.039	0.1005
Parasomnia	0.58 (0.16)	0.0003	**0.0031**
Sleep anxiety	0.35 (0.16)	0.0295	0.0869
Sleep duration	0.2 (0.13)	0.116	0.1826
Sleep onset delay	0.06 (0.06)	0.3143	0.4019

Point estimates (Standard errors), p-values, and false discovery rates for the effect of presence of GI symptoms from linear mixed effect models relating behavior scores to the presence of GI symptoms (Y/N) + Group (ASD/TD) + sex + Age + IQ (FSIQ) + Random Subject. Bolded cells indicate FDR < 0.05.

#### GIS frequency, as indexed by number of concurrent GIS, and behavioral scores

Considering only participants within the ASD group, increasing number of GIS was significantly associated with increased impairment for 22 behavioral domains of the 41 behavioral and developmental scores evaluated (see Table 5). Specifically, scores were significantly elevated for those experiencing a greater number of concurrent GIS and (1) compulsive/ritualistic/sameness behaviors (p = 0.03), and total score subscales based on the RBS (p = 0.03); (2) repetitive and restrictive behaviors (p = 0.03), social communication and interaction (p = 0.01), and total score subscales on the SRS (p = 0.012); (3) auditory filtering (p = 0.01), tactile sensitivity (p = 0.03), taste/smell sensitivity (p = 0.01), visual/auditory sensitivity (p = 0.01), and total score on the SSP-1 (p = 0.003); (4) anxious/depressed (p = 0.004), emotional reactivity (p = 0.03), internalizing behavior (p < 0.0001), sleep problems (p = 0.009), and somatic complaints (p < 0.0001) subscales on the CBCL; and (5) disordered breathing (p = 0.001), daytime sleepiness (p = 0.013), night walking (p = 0.012), parasomnia (p < 0.0001), sleep anxiety (p = 0.006), sleep duration (p = 0.02), and total score subscales (p = 0.001) on the CSHQ (Table 5). There were no significant differences on any of the VABS scores. Full model results are provided in Supplementary Tables 5(a) and (b).

**Table 5.** Association between number of concurrent GIS and behavioral scores.

	Estimate (SE)	p	FDR
**Repetitive Behavior Scale Total Score**	1.46 (0.58)	0.0131	**0.0302**
Compulsive/ritualistic/sameness behaviors	0.93 (0.38)	0.0152	**0.0326**
Self-injurious behaviors	0.22 (0.1)	0.0415	0.0727
Restrictive stereotyped behaviors	0.3 (0.18)	0.0972	0.1431
**Social Responsiveness Scale-2 Total Score**	1.07 (0.36)	0.0035	**0.0128**
Repetitive and restrictive behaviors	1.07 (0.44)	0.0176	**0.034**
Social Communication and Interaction	1.05 (0.34)	0.0027	**0.011**
**Short Sensory Profile-1 Total Score**	-3.12 (0.84)	0.0005	**0.0036**
Auditory filtering	-0.52 (0.18)	0.0066	**0.0169**
Low energy/weak	-0.47 (0.23)	0.0444	0.0727
Movement sensitivity	-0.05 (0.1)	0.6485	0.6984
Tactile sensitivity	-0.43 (0.19)	0.0239	**0.0449**
Taste/smell sensitivity	-0.65 (0.22)	0.0045	**0.0133**
Under-responsive/seeks sensation	-0.35 (0.24)	0.1434	0.1973
Visual/auditory sensitivity	-0.53 (0.18)	0.0049	**0.0133**
**Child Behavior Checklist**			
Externalizing problems	0.37 (0.34)	0.2859	0.3357
Internalizing problems	1.62 (0.31)	<0.0001	**<0.0001**
Aggressive behavior	0.26 (0.3)	0.3864	0.4394
Anxious/depressed	0.76 (0.22)	0.0007	**0.0043**
Attention problems	0.58 (0.28)	0.0443	0.0727
Defiant behavior	-0.1 (0.4)	0.8191	0.8404
Emotionally reactive	0.79 (0.32)	0.0167	**0.034**
Sleep problems	1.09 (0.34)	0.0021	**0.0095**
Social problems	1.36 (0.56)	0.0584	0.0925
Somatic complaints	2.48 (0.22)	<0.0001	**<0.0001**
Thought problem	0.59 (0.64)	0.429	0.4764
Withdrawn	0.45 (0.33)	0.1669	0.2207
**Children’s Sleep Habit Questionnaire Total Score**	1.16 (0.29)	<0.0001	**0.001**
Bedtime resistance	0.19 (0.12)	0.1175	0.1652
Disordered breathing	0.09 (0.02)	0.0002	**0.0019**
Daytime sleepiness	0.3 (0.1)	0.0046	**0.0133**
Night waking	0.17 (0.06)	0.0037	**0.0128**
Parasomnia	0.37 (0.07)	<0.0001	**<0.0001**
Sleep anxiety	0.23 (0.07)	0.0012	**0.006**
Sleep duration	0.15 (0.06)	0.012	**0.0291**
Sleep onset delay	0.03 (0.03)	0.2647	0.3196

Point estimates (Standard errors), p-values, and false discovery rates for the effect of presence of the number of GI symptoms from linear mixed effect models relating behavior scores to GI severity (Count) + Sex + Age + IQ + Random Subject. Bolded cells indicate FDR < 0.05.

## Discussion

This study represents the first analysis of longitudinal GIS from early to middle childhood utilizing strict GI classification criteria obtained via parent interview with a developmental pediatrician and use of standardized measures in children with autism and typically developing controls. GIS are frequently reported by parents/caregivers of children with autism but are often misdiagnosed or under-reported in clinical settings due to associated communication and sensory challenges in this population. Similarly, parental uncertainty on symptoms and potential health inequalities for autistic individuals to access the right medical services due to patient–provider communication issues, medical staff’s lack of knowledge regarding autism, judgment, and lack of adaptability in the medical environment (Douglas et al., 2023) may affect the timely identification and treatment of GIS. Our findings support the hypothesis that children with autism experience more frequent and persistent GIS of unknown clinical etiology than their TD counterparts, even when using a rigorous clinical classification. Almost half (47%) of the children in the ASD group experienced GIS at one time point between 2 and 12 years of age, 40% experienced GIS at two time points, and for 30% it continued through all three time points. The RR of GIS is higher for children with autism supporting previous studies indicating a higher probability of experiencing GIS (Chaidez et al., 2014; Holingue et al., 2018; McElhanon et al., 2014; Restrepo et al., 2020). Moreover, children in the ASD group presented with a higher number of concurrent GIS at each time point compared to the TD group. The most reported symptoms included constipation, diarrhea, gaseousness/bloating, and abdominal pain similar to previously published studies (Buie et al., 2010; Holingue et al., 2018; McElhanon et al., 2014). These findings indicate that children with autism are more likely to experience persistent symptomatology throughout their childhood.

This study also provided support for the hypothesis that the presence of GIS is associated with the frequency and degree of challenging behaviors (Ferguson et al., 2019; Mazefsky et al., 2014; Prosperi et al., 2017). Children with persistent GIS in both groups presented with more severe behavioral scores across all domains measured. Children with autism experiencing GIS presented higher autistic features, internalizing problems, sleep issues, and somatic complaints, which may reflect physical symptoms associated with medical or mental health problems. Moreover, those experiencing multiple concurrent GIS presented with significantly worse behavioral scores including measures of social communication, repetitive behaviors, sensory processing, behavioral, and sleep issues, suggesting a greater impact for those participants experiencing multiple GIS. These findings support available literature suggesting that GIS may be linked to more impairing behaviors in children with autism compared to children without autism (Aldinger et al., 2015; Ferguson et al., 2017, 2019; Maenner et al., 2012; Restrepo et al., 2020). Therefore, the emergence of new behavioral issues or the exacerbation of existing ones should alert families and medical providers about the possibility that an underlying GI problem is also present and needs to be explored as a potential explanation for the increased behavioral challenges.

Our findings are similar to those previously reported by two longitudinal studies evaluating the presence and persistence of GIS in children with autism at 1 (Mazurek et al., 2014) and 2 years from baseline (Mannion et al., 2013); these previous studies have also used a GI questionnaire to evaluate the presence and persistence of symptomatology. Although other retrospective analysis of longitudinal data based on medical records or database retrieval reported no difference in the incidence of GI concerns between individuals with and without ASD across the lifespan, findings are likely influenced by methodological issues including a retrospective data collection through diagnostic codes available in medical records instead of using a systematic way to document the presence of GIS in children with autism limiting the generalization and comparison of their findings (Black et al., 2002; Ibrahim et al., 2009; Mouridsen et al., 2010).

This study had several strengths including a diverse sample, longitudinal design, and well-characterized sample. The presence of GIS was evaluated by trained medical specialist, and the GI status was defined by two independent physicians. Careful consideration was given to assess only GIS that were not associated with a diagnosed underlying GI condition explaining those symptoms via clinical consensus. The underlying cause of GIS experienced by children in this study is currently unknown. There were also several limitations. First, GIS information was obtained from parent-reported questionnaire that was not subject to reliability or validity testing but derived from a set of items developed to assess these issues in children with autism which has been used by previous studies (Chaidez et al., 2014; Restrepo et al., 2020); this questionnaire inquires about the presence of the most common GIS overtime and other symptoms frequently reported in the children with autism including food allergies, food sensitivities, and other underlying diagnoses in open-ended fashion. Moreover, there is no consensus about the optimal measure of GIS in this population, the psychometric properties of most assessment tools previously used by other studies are unknown, and there is no single GI measure to reliably assess the presence of GIS in each autistic individual. Second, our study sample was ascertained in a single location in the United States that limits generalizability to the US population. Third, although our analysis has a relatively large longitudinal dataset, the number of participants decreased at subsequent time points, impacting our statistical power and raising concerns about whether longitudinal data was missing at random, though our comparison of baseline characteristics of participants in the study compared to those that did not have subsequent longitudinal data did not reveal any systematic differences in IQ or autism characteristics or proportion endorsing GIS.

Nevertheless, the current study findings have clear implications for future research and clinical practice. Results support the need for future research to assess the presence of and persistence of GIS in children with autism throughout childhood, including factors during early childhood that may influence the persistence of GIS in the absence of a clear etiology, and how to best identify those at risk of chronic GIS. Although this study does not investigate the underlying cause of GIS, our findings strongly support further efforts to understand the phenotypical heterogeneity in the autistic population and to determine whether GI problems correlate with specific presentations influencing behavioral presentations, sleep, feeding patterns, and GI dysfunction.

With regard to clinical practice, there is a clear need to increase awareness of risk for GIS in children with autism and best practices of care. We found that GIS are frequent and tend to persist throughout childhood in the ASD group studied. Thus, when parents have concerns about GIS in children with autism, pediatric providers should screen for possible GIS and manage and/or refer for care in a timely fashion when indicated. Similarly, when parents raise concerns about a new behavior problem arising, it is important to screen for GIS, as children with autism may not be able to report their symptoms at all and parent observation is the most important source of information. Finally, it is important to keep in mind that the GI problems of children with autism may be difficult to identify due to challenges in communication and sensory processing, more specifically interoception. If unrecognized and untreated, GIS can negatively influence their behavior, daily functioning, and psychological and physical well-being at home and at school.

## Supplemental Material

sj-docx-1-aut-10.1177_13623613251362349 – Supplemental material for A longitudinal evaluation of gastrointestinal symptoms in children with autism spectrum disorder Supplemental material, sj-docx-1-aut-10.1177_13623613251362349 for A longitudinal evaluation of gastrointestinal symptoms in children with autism spectrum disorder by Bibiana Restrepo, Sandra L Taylor, Matthew Dominic Ponzini, Kathleen Angkustsiri, Marjorie Solomon, Sally J Rogers, Paul Ashwood, Daphne S Say, Sonny Caceres, Shayan Alavynejad, Brianna Heath, David G Amaral and Christine Wu Nordahl in Autism
