# Supplementary material for: A longitudinal evaluation of gastrointestinal symptoms in children with autism spectrum disorder
Source: Autism. 2025 Aug 28;29(11):2832–45. doi: 10.1177/13623613251362349 (PMC12404668; doi:10.1177/13623613251362349)
Supplement: sj-docx-1-aut-10.1177_13623613251362349 – Supplemental material for A longitudinal evaluation of gastrointestinal symptoms in children with autism spectrum disorder [file sj-docx-1-aut-10.1177_13623613251362349.docx]

|  | Count | % |
| --- | --- | --- |
| Mother | 419 | 88.2% |
| Father | 45 | 9.5% |
| Both Parents | 3 | 0.6% |
| Grandparent | 3 | 0.6% |
| Not reported | 5 | 1.1% |

**Supplemental Table 1**. Breakdown of caregiver reporting for parent questionnaires utilized in this study. Data from the Vineland Adaptive Behavior Scales questionnaire at Visit 1 is provided. Families are instructed to have a caregiver that resides with the child and knows their behaviors well to complete the parent questionnaires.

|  | ASD | | | | | TD | | | | |
| --- | --- | --- | --- | --- | --- | --- | --- | --- | --- | --- |
|  | **Overall** | **Returned, GI Data** | **Returned, Missing GI Data** | **Attrition** | **p-value^2^** | **Overall** | **Returned, GI Data** | **Returned, Missing GI Data** | **Attrition** | **p-value**^2^ |
| n | 283 | 91 | 93 | 99 |  | 128 | 53 | 39 | 36 |  |
| IQ FSIQ^1^ | 61.1  (46.0, 76.8) | 63.6  (49.0, 77.7) | 60.2  (43.7, 73.1) | 59.5  (44.9, 80.0) | 0.430 | 105.4 (98.0, 115.3) | 106.5 (98.6, 115.2) | 102.5 (97.7, 118.2) | 105.7 (96.3, 115.8) | 0.828 |
| ADOS CSS^1^  Score | 7.0  (6.0, 9.0) | 7.0  (6.0, 9.0) | 8.0  (6.0, 9.0) | 7.0  (6.0, 9.0) | 0.206 | -- | -- | -- | -- | -- |
| GIS |  |  |  |  | 0.189 |  |  |  |  | 0.284 |
| No | 119 (43%) | 44 (51%) | 38 (42%) | 37 (37%) |  | 86 (68%) | 39 (74%) | 22 (58%) | 25 (71%) |  |
| Yes | 158 (57%) | 43 (49%) | 53 (58%) | 62 (63%) |  | 40 (32%) | 14 (26%) | 16 (42%) | 10 (29%) |  |
| ^1^Median (Q1, Q3); n (%) | | | | | | | | | | |
| ^2^Kruskal-Wallis rank sum test; Fisher's exact test | | | | | | | | | | |

**Supplemental Table 2**. Summary of baseline Visit 1 characteristics by Visit 3 Status for ASD and TD groups Returned, GI Data = participants who are included in the current study; Returned, Missing GI Data = Returned for study visits but missing GI data; Attrition = lost to follow up.

|  | **ASD** | | | | | **TD** | | | | |
| --- | --- | --- | --- | --- | --- | --- | --- | --- | --- | --- |
|  | **Overall** | **Returned, GI Data** | **Returned, Missing GI Data** | **Attrition** | **p-value**^2^ | **Overall** | **Returned, GI Data** | **Returned, Missing GI Data** | **Attrition** | **p-value**^2^ |
|  | 191 | 77 | 62 | 52 |  | 90 | 51 | 18 | 21 |  |
| IQ-FSIQ^1^ | 59.8 (45.0, 73.8) | 59.4 (46.0, 76.6) | 59.2 (43.2, 74.9) | 61.2 (48.4, 71.4) | 0.849 | 104.7 (98.0, 115.3) | 105.4 (98.2, 115.3) | 108.8 (97.7, 118.2) | 102.5 (98.0, 111.5) | 0.785 |
| ADOS CSS^1^ | 8.0 (6.0, 9.0) | 7.0 (6.0, 8.0) | 8.0 (6.0, 8.0) | 8.5 (7.0, 10.0) | 0.055 | -- | -- | -- | -- |  |
| GI symptoms |  |  |  |  | 0.359 |  |  |  |  | 0.746 |
| 0 | 86 (46%) | 37 (49%) | 31 (50%) | 18 (38%) |  | 60 (68%) | 35 (71%) | 12 (67%) | 13 (62%) |  |
| 1 | 99 (54%) | 38 (51%) | 31 (50%) | 30 (63%) |  | 28 (32%) | 14 (29%) | 6 (33%) | 8 (38%) |  |
| ^1^Median (Q1, Q3); n (%) | | | | | | | | | | |
| ^2^Kruskal-Wallis rank sum test; Fisher's exact test | | | | | | | | | | |

**Supplementary Table 3**. Summary of baseline Visit 1 characteristics by Visit 4 status for ASD and TD groups. Returned, GI Data = participants included in analyses; Returned, Missing GI Data = Returned for study visits but missing GI data; Attrition = lost to follow up.

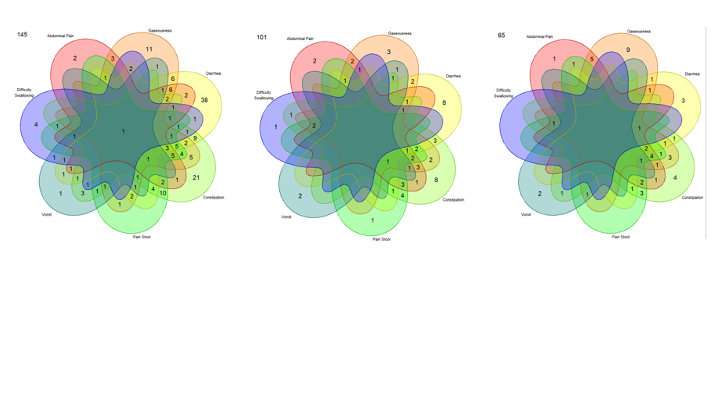


**Supplemental Figure 1**. Venn diagrams showing the seven most commonly reported GI symptoms and their intersection; abdominal pain in red, difficulty swallowing in purple, vomiting in blue, pain on stooling in green, constipation in lime, diarrhea in yellow and gaseousness represented in orange in children in the ASD group at Visits 1, 3, and 4 respectively.
